# Psychophysiological characterization of different capoeira performances in experienced individuals: A randomized controlled trial

**DOI:** 10.1371/journal.pone.0207276

**Published:** 2018-11-15

**Authors:** Sérgio R. Moreira, Alfredo A. Teixeira-Araujo, Eduardo S. Numata Filho, Milton R. Moraes, Herbert G. Simões

**Affiliations:** 1 Graduate Program on Physical Education, Federal University of Vale do São Francisco–UNIVASF, PE, Petrolina, Brazil; 2 Graduate Program Health and Biological Sciences, Federal University of Vale do Sao Francisco–UNIVASF, PE, Petrolina, Brazil; 3 College of Physical Education, UNILEÃO–University Center, Juazeiro do Norte–CE, Brazil; 4 Graduate Program on Physical Education of Catholic University of Brasilia–UCB, DF, Brasília, Brazil; University of Essex, UNITED KINGDOM

## Abstract

The aim of this study was to characterize the psychophysiological demand in different capoeira performances. Eleven experienced capoeira practitioners underwent the following procedures in different days: 1) anamnesis and anthropometric measurements; 2) aerobic performance assessment; 3 to 5) performances of 90 seconds in three capoeira styles (*Angola*, *Benguela* and *São Bento*), which were performed in randomized controlled cross-over design. The psychophysiological demand was assessed through the heart rate (HR), R-R interval (RRi), blood pressure (BP), blood lactate ([Lac]), blood glucose ([Gluc]), rating perceived effort (RPE), feeling scale (FS) and perceived activation (PA). Descriptive statistics with mean and standard deviation was performed. A two-way repeated measures ANOVA with Bonferroni post-hoc test was used. The *Angola* demand was between 56–71% maximal HR with [Lac]_PEAK_: 6.9±2.9 mM, RPE_PEAK_: 10.0±2.2 pts and FS: 2.7±1.7 pts, while in the *Benguela* participants reached 64–85% maximal HR with [Lac]_PEAK_: 9.5±3.0 mM, RPE_PEAK_: 11.0±2.8 pts and FS: 2.1±1.6 pts and for *São Bento* between 69–102% maximal HR with [Lac]_PEAK_: 15.7±2.6 mM, RPE_PEAK_: 15.5±4.1 pts and FS: -0.8±3.0 pts. Interaction time*performance occurred to [Lac] (F = 42.157; p<0.001), HR (F = 12.154; p<0.001), RRi (F = 4.774; p<0.001), parasympathetic modulation-rMSSD (F = 3.189; p<0.01), [Gluc] (F = 2.152; p<0.05), RPE (F = 5.855; p<0.01), FS (F = 2.448; p<0.05) and PA (F = 3.893; p<0.05). We concluded that *São Bento* rhythm resulted in a greater physiological demand according to the HR, [Lac] and cardiac autonomic indicators, with the *Benguela* rhythm being intermediate while the *Angola* rhythm presented a reduced demand. The [Gluc] increased after the *São Bento* rhythm when compared to the other rhythms. The perceptual demand presented increased in terms of RPE and PA variables and decreased in terms of FS when the *São Bento* performance was analyzed in comparison to the *Angola* and/or *Benguela* in capoeira.

## Introduction

Defined as an athletic performance involving an attack and defense system with individual characteristics, capoeira is considered a martial art that is genuinely Brazilian and one of the most important popular sports manifestations in the country [1]. In addition to its sporty character, capoeira was recognized by UNESCO (*United Nations Educational*, *Scientific and Cultural Organization)* in 2014 as an Intangible Humanity Heritage and nowadays is practiced in more than 150 countries across five continents [2] and by various social groups [3]. The traditional capoeira styles are grounded in *Capoeira Angola* by Master Pastinha and *Capoeira Regional* by Master Bimba, and the basic moves in the modality are made up of both styles [4].

Among the existing capoeira styles, different performances (rhythms) are developed, which are characterized by *Angola*, *Benguela* and *São Bento Grande* rhythms. Silva et al. [5] observed that the *Angola* performance is characterized by a slower rhythm. According to these authors, *Benguela* performances involve intermediate rhythm, characterized by a greater volume of creative movements, played at a faster cadence than that in *Angola* performance. In *São Bento Grande* performances, the practitioner develops the movement at a more accelerated rhythm compared to the previous aforementioned performances, and its practice is marked by harder physical movements in general and greater muscle power in the movements of attack.

Our laboratory has demonstrated that the capoeira practice can chronically result in improvements of heart rate, cardiac autonomic indices [6] and angular flexibility [7], which are associated with cardiovascular health [8] and functional performance [9] of the practitioners. However, no systematic research has sought to highlight the physiological and metabolic responses after different capoeira performances. Therefore, from the beginning of the scientific findings regarding capoeira*’s* effects on physiological variables [5–7,10], the need to characterize the physiological demand of this modality has been seen. Thus, blood lactate responses as a primary marker of intensity [11], followed by secondary variables such as heart rate and cardiac autonomic modulation indicators [12], could suggest what intensity domains and physiological requirements are predominant during different capoeira rhythms, which would aid in the understanding of previous findings [6], as well as hypotheses for future studies in this modality.

Furthermore, the perceptual characteristics during the execution of various exercise methods have been recent objects of study [13,14], and research regarding the nature of the feelings in terms of the particular stress conditions has become relevant [15]. Hence, verifying the capoeira perceptual demand could contribute significantly to the interpretation of the psychophysiological integration and especially different rhythms imposed in this modality, which consequently could justify responses obtained with the training [6,7].

In addition, different capoeira energy demands can be generated due to the amount of explosive movements (such as kicks, jumps and spins) that are characteristic of the modality, which, in addition to the execution speed of different rhythms, still require various degrees of difficulty from the practitioner. Thus, understanding the psychophysiological characterization of different capoeira performances could contribute to better planning of physical training strategies toward the specificity required in the modality, as well as to the safety analysis when applying capoeira to populations with some health risk factors because the intensities can vary significantly among the different rhythms mentioned. However, there is still no quantification regarding such responses in the psychophysiological and metabolic aspects. In this sense, the objective of this study was to characterize the psychophysiological demand of different capoeira performances on male individuals experienced in the modality.

## Materials and methods

### Subjects and study design

The present study was approved by the Research Ethics Committee from the UNIVASF under number 1.687.330 and was enrolled in *ClinicalTrials*.*gov* from NCT03170921. A randomized controlled cross-over study design was performed. Healthy adults with experience in capoeira were investigated from five visits to the Physical Education Department at the Federal university of Vale do São Francisco–UNIVASF.

**Fig 1:**
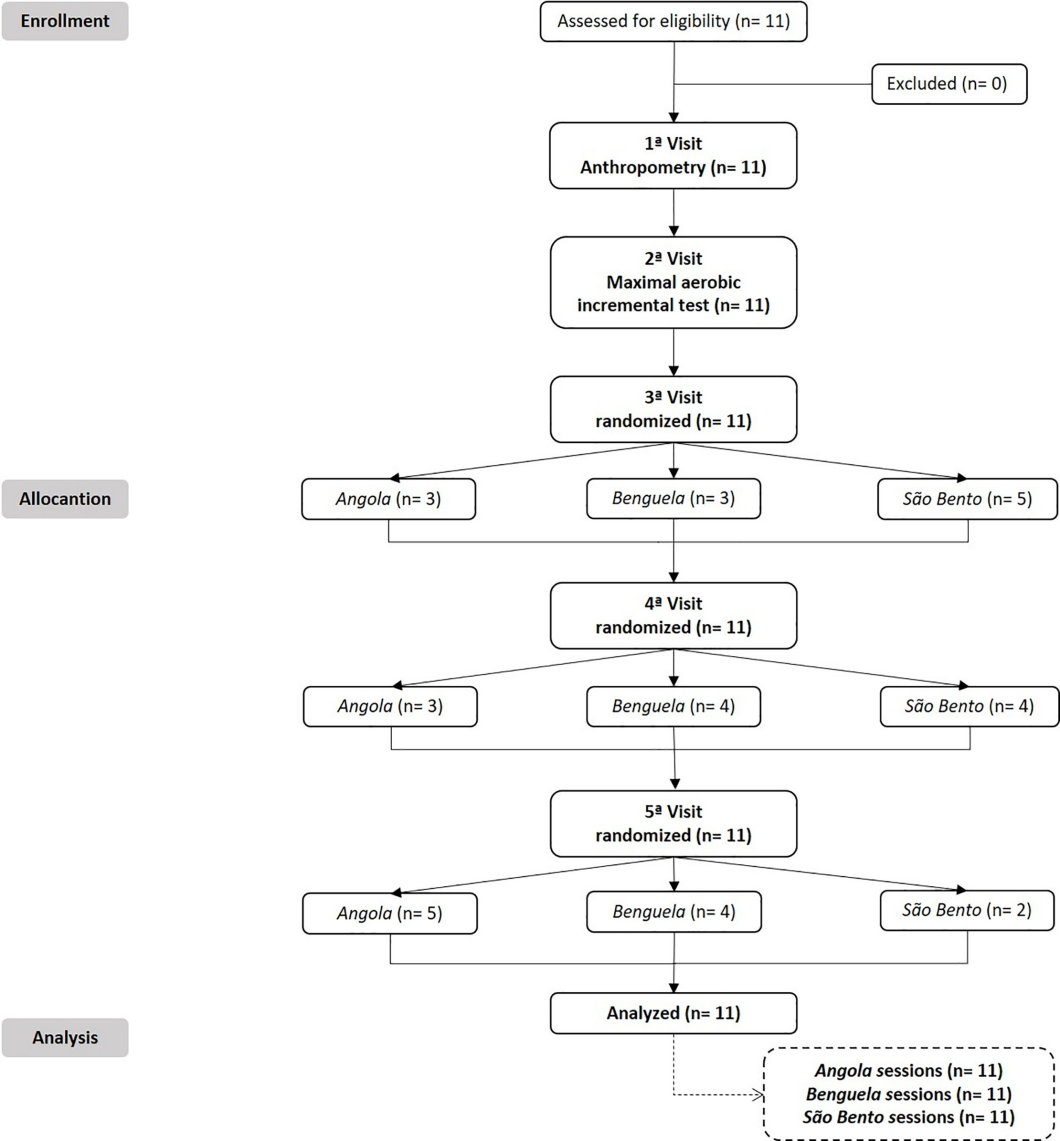
Consort flow diagram. After the first two visits, all participants performed in randomized controlled cross-over design with three experimental sessions in different capoeira rhythms (*Angola*, *Benguela* and *São Bento*).

The recruitment of volunteers occurred in the first week of September/2016 and the laboratory evaluations and protocol experimental occurred in the second and third week of September/2016. The study had as inclusion criteria: (i) to be male between 18 and 40 years; (ii) a capoeira practitioner for at least 2 years; and (iii) mastering technique of the three rhythms of performance investigated (*Angola*, *Benguela* and *São Bento*). As exclusion criteria: (i) to be hypertensive, diabetic or obese; (ii) a smoker; (iii) alcohol addicted and (iv) have some cardiovascular dysfunction or musculoskeletal problem. The participants volunteered and signed a written informed consent in accordance with resolution 466/12 from the National Health Council of Brazil and principles of the Declaration of Helsinki. All volunteers recruited satisfied the inclusion criteria, no one had excluded and were, in crossover random order, performed the three rhythms investigated (Fig 1). It should be noted that neither the participants nor the researchers were blinded for interventions and/or analyses. So, the sample was composed by 11 male individuals with experience in the three rhythms of capoeira performance selected for the study. It is highlighted that the sample was considered as ‘‘recreationally trained”, defined as completing regular bouts of exercise (30 min or more, three times per week) in a non-competitive setting for at least 36 months before recruitment. The general and functional characteristic of the sample are presented in Table 1.

**Table 1:** Mean (±SD) of general characteristics of the sample.

N	11
Practice time (y)	10.7±5.8
Age (y)	33.0±7.3
Weight (kg)	75.6±13.5
Height (cm)	173.3±5.2
Body mass index (kg·m^-2^)	25.1±3.8
Waist circumference (cm)	84.0±9.3
Skinfold ∑7 (mm)	71.7±28.0
Body fat (%)	15.0±5.4
Resting SBP (mmHg)	123.0±12.3
Resting DBP (mmHg)	80.4±8.9
Postprandial glucose (mM)	6.2±0.7
PPO_MAIT_ (watts)	266.0±45.0
HR_PPO_ (bpm)	188.0±11.0
RPE_PPO_ (Borg_[__6__–__20__]_)	18.8±1.4

**SBP**: systolic blood pressure; **DBP**: diastolic blood pressure; **PPO**: peak power output; **MAIT**: maximal aerobic incremental test; **HR**: heart rate; **RPE**: rate of perceived exertion. The Skinfold ∑7 were performed from triceps, subscapularis, suprailiac, abdomen, chest, thigh and calf measures using a traditional scientific skinfold caliper (CESCORF/Mitutoyo, Porto Alegre/RS, Brasil). The body fat was calculated by predictive equations.

**Fig 2:**
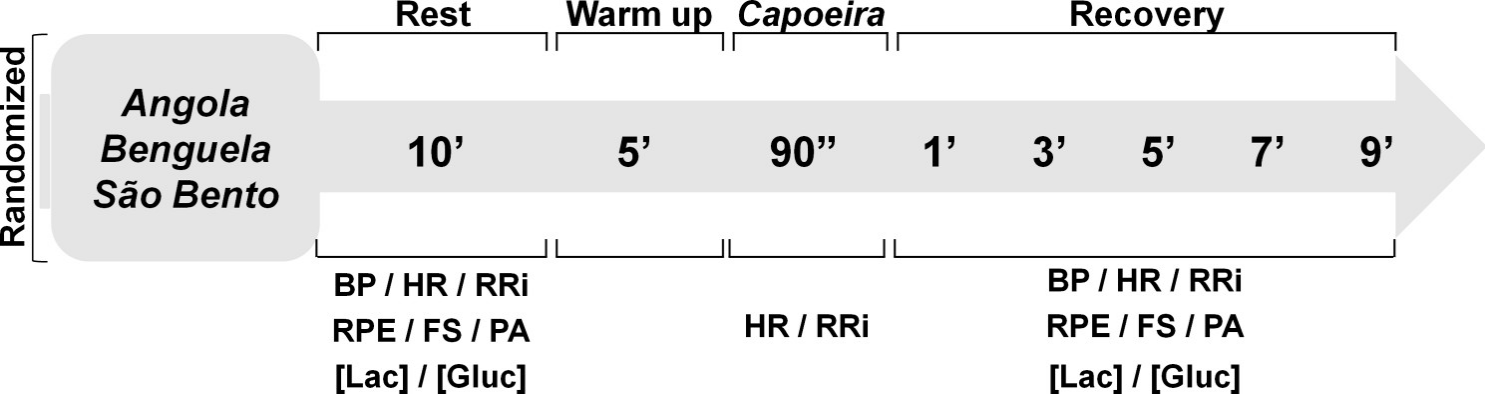
Experimental design. BP: blood pressure; HR: heart rate; RRi: R-R interval (heart rate variability); RPE: rate of perceived exertion; FS: feeling scale; PA: perceived activation; [Lac]: blood lactate concentration; [Gluc]: blood glucose concentration.

In the firsts two visits anthropometric characteristics and aerobic performance of the participants were evaluated. In the next three visits in different days and a random order, the participants performed the experimental sessions in different rhythms of capoeira performance (*Angola*, *Benguela* and *São Bento*). The Fig 1 shows a crossover random design utilized in the present study. In the first experimental session (3^a^ visit), three participants performed the *Angola* rhythm, while three performed the *Benguela* rhythm and five participants performed the *São Bento* rhythm. In the second experimental session (4^a^ visit), three volunteers performed the *Angola* rhythm, four performed the *Benguela* rhythm and four volunteers performed the *São Bento* rhythm. In the third experimental session (5^a^ visit), five volunteers performed the *Angola* rhythm, four the *Benguela* rhythm and five volunteers performed the *São Bento* rhythm. Thus, the sample was composed by 11 participants, who randomly performed every studied rhythms (*Angola*, *Benguela* and *São Bento*). Measurements of physiological demand (heart rate—HR, cardiac autonomic modulation, blood pressure—BP, blood lactate—[Lac], blood glucose—[Gluc]) and perceptual variable (rate of perceived effort—RPE, feeling scale—FS and perceived activation—PA) were performed at rest and during recovery after the different rhythms of capoeira performance. Fig 2 presents the experimental design.

### Maximal aerobic incremental test (MAIT)

For the evaluation of aerobic fitness a MAIT in the cyclergometer was performed (Biotec 2100, Cefise). MAIT began with 50 watts followed by increments of 25 watts at each 1-min stage. The participants were instructed to maintain a frequency of 50 rpm until voluntary exhaustion or not being able to maintain the pre-established rpm [16]. The last stage of the MAIT was considered valid when the individual remained at least 50% of the stage time duration in exercise. So, the power output, HR and RPE peak measures were obtained in the last stage.

### Capoeira experimental sessions

The pairs of participants were matched based on weight, height and technical level and then were randomly assigned, by sort paper, to realize the capoeira performance experimental sessions based on three distinct rhythms, namely: 1) *Angola*; *2*) *Benguela* and; *3*) *São Bento*. The performance rhythms were performed in different days and separated by an interval of 48 hours. All the performance rhythms were accompanied by standardized percussion instruments, with three *berimbaus*, one with severe intonation (*berimbau gunga*), another intermediate (medium *berimbau*) and the last with acute intonation (*berimbau viola*), an *atabaque* and two tambourines. The musical instruments were played by experienced individuals, keeping the same players for each performance (pairs of participants), and the songs were also sung in a standardized way for each performance. The performances in different rhythms always occurred at the same time of day between 3:00 and 4:00 p.m.

A period of rest (pre-intervention) with a duration of 10 minutes occurred prior to completion of each performance rhythm, with volunteers being seated comfortably in an upholstered armchair. At the end of the rest measurements of the physiological and the perceptual variables of the study were performed. Soon after the pre-intervention a standardized warm-up/stretching in light intensity perceived was performed during 5 minutes with the own capoeira movements. Then pairs of participants were conducted for the intervention with the performing of its respective capoeira performance rhythms (*Angola*, *Benguela* or *São Bento*). Each rhythm of performance had duration of 90 seconds and participants received voice instructions to start and finish the performance. The selected duration is justified from average performance time of rhythms analyzed during world competitions, which are approximately 165, 60 and 45 seconds of performance for *Angola*, *Benguela* and *São Bento*, respectively. After each physical performance with the capoeira rhythm, during recovery period (post- intervention) in the moments 1, 3, 5, 7, and 9 minutes was measured the physiological and the perceptual variables of the study. The pre-intervention, intervention and post-intervention were performed in a room free of noise and temperature between 22–24°C.

### Physiological variables

During the experimental sessions the participants used a device brand Polar Electro mod. RS800CX duly validated [17] to record the HR and R-R intervals (RRi) of HR variability (HRV). In addition to the analysis pre- and post-intervention, during the intervention it was considered the lowest HR and RRi (_MIN_), the average HR and RRi (_AVE_) and the largest HR and RRi (_PEAK_). The records of the RRi, during the intervention, were exported from the device Polar to the software Polar ProTrainer 5 by infrared and analyzed through the software Kubios HRV version 2.0. In order not to jeopardize the reliability of the indexes obtained, the early ectopic and artifact beats were removed and adjusted, being a criterion the differences greater than 20% in relation to adjacent beats. After editing the RRi, analyzes using linear methods in the time and frequency domain were performed with the objective of estimation of cardiac autonomic modulation. Concerning the time domain, the parameters analyzed were the absolute mean RRi and rMSSD (root mean square differences of successive RRi) as an indicator of the modulation of the parasympathetic nervous system. As to frequency domain the high frequency components (HF: 0.15 to 0.4 Hz) as well as the low frequency (LF: 0.04 to 0.15 Hz) were analyzed to calculate the LF:HF ratio, which characterizes the cardiac sympathovagal balance [18].

In the pre- and post-intervention BP measures were conducted by means of an automatic sphygmomanometer brand Microlife, mod. BP3AC1-1PC, equipment validated and with high reproducibility for normotensive individuals [19]. The procedures adopted for BP evaluation were those recommended by the Cardiology Brazilian Society [20].

Besides, in the pre- and post-intervention periods a puncture in the participants’ ear lobe was done to collect 25 μl of capillary blood by calibrated capillaries. The blood samples were deposited in *Eppendorf* microtubules and stored in 50 μl of sodium fluoride to 1% for later analysis of [Lac] and [Gluc]. The [Lac] and [Gluc] was measured by an electro-enzymatic biochemistry analyzer (Yellow Springs 2.700 STAT, OH, USA).

### Perceptual variables

#### Rate of perceived exertion (RPE)

The whole-body perceived exertion was assessed using the Borg’s RPE [6–20] Scale [21]. Before the MAIT, and experimental sessions with capoeira, the meaning of RPE was explained to the subjects. A rating of 6 (low anchor, “very, very light”) was assigned to the lowest exercise intensity, while a rating of 20 (high anchor, “very, very hard”) was assigned to the highest exercise intensity.

#### Basic affective response

The Feeling Scale (FS) was used to assess the affective response and it is an 11-point bipolar scale ranging from +5 to -5, commonly used to measure pleasure/displeasure during exercise [13]. This scale presents the following verbal anchors: -5 = very bad; -3 = bad; -1 = fairly bad; 0 = neutral; +1 fairly good; +3 = good; and +5 = very good. The subjects received standard instructions regarding to the use of the FS in according to Hardy and Rejeski [22].

#### Perceived activation (PA)

The PA scale was used to assess the arousal state of the participants [23]. The PA scale is a 6-point single-item measure, ranging from +1 (low arousal) to +6 (high arousal).

In addition, an integrated analysis interpolating FS and PA responses was performed, aiming to obtain the sense that the affective domain points from different quadrants of the circumplex model (high activation-pleasure; high activation-displeasure; low activation-displeasure; low activation-pleasure) [24].

Finally, standard definitions of perceptual responses and separate instructional sets for scales were read to the participants immediately before the MAIT. The low and high perceptual anchors for the RPE, FS and PA scale were established during the MAIT. RPE, FS and PA values were evaluated randomly during the last 10 seconds of each stage of MAIT, during pre-intervention and, different moments of post-intervention.

### Statistical analysis

Descriptive statistics with mean and standard deviation was performed. The normality of data distribution was tested by the Shapiro-Wilk test. ANOVA two-way for repeated measures, reporting the “F-ratio", degrees of freedom and the "P" value was used to verify the interaction time*rhythms and the main effect of time within each capoeira performance. The test of Mauchly was adopted to verify the sphericity of the data, which, in case of violation, the degrees of freedom would be corrected by the epsilon of Greenhouse-Geisser. Partial eta squared (η_p_^2^) was used to determine the effect size. Post hoc of Bonferroni was adopted for identification of pairs of difference. Considering the sample size of this study and an alpha error of 0.05, the mean statistical power (1 –β) of the analyses as presented in Tables 2 and 3 were 0.99 and 0.95, respectively. The alpha was set at 5% and the software used was SPSS 22.0 (SPSS, Inc., Chicago, IL).

**Table 2:** Mean (±SD) of heart rate variability responses (RRi) in the minimum heart rate (HR_MIN_), average heart rate (HR_AVE_) and peak heart rate (HR_PEAK_) during different capoeira performances in absolute (bpm) and relative values to the heart rate of the end of the maximal aerobic incremental test equivalent to the peak power output (%HR_PPO_) (n = 11).

	*Angola*	*Benguela*	*São Bento*
**HR**_**MIN**_			
** (bpm)**	105 ± 12*^a^	119 ± 8^†^^a^	129 ± 5^a^
**(%HR**_**PPO**_**)**	56 ± 7*^a^	64 ± 5^†^^a^	69 ± 3^a^
**RRi (ms)**	606 ± 79*^a^	526 ± 74^†^^a^	466 ± 18^a^
**HR**_**AVE**_			
** (bpm)**	126 ± 21*^b^	153 ± 9^†^^b^	178 ± 8^b^
**(%HR**_**PPO**_**)**	67 ± 12*^b^	82 ± 6^†^^b^	95 ± 3^b^
**RRi (ms)**	519 ± 97*^b^	415 ± 66^†^^b^	341 ± 16^b^
**HR**_**PEAK**_			
** (bpm)**	132 ± 19*	158 ± 9^†^	191 ± 12
**(%HR**_**PPO**_**)**	71 ± 11*	85 ± 6^†^	102 ± 3
**RRi (ms)**	489 ± 82*	401 ± 67^†^	318 ± 19

*P<0.01 to *Benguela* and *São Bento*

†P<0.01 to *São Bento*

^a^P<0.01 to RRi-HR_AVE_ and RRi-HR_PEAK_ in the same performance

^b^P<0.01 to RRi-HR_PEAK_ in the same performance.

**Table 3:** Mean (±SD) of psychophysiological variables in the resting (Rest) and during recovery minutes (Rec1’ to Rec9’) after different capoeira performances (n = 11).

		Rest	Rec1’	Rec3’	Rec5’	Rec7’	Rec9’	Main effect	
								Time	Time*Rhythm
**HR (bpm)**	***Angola***	79±8	109±15*	95±12*	92±12*	92±11*	91±11*	**F = 88.026** **P < 0.001** ***η***_***p***_^***2***^ **= 0.90**	**F = 12.154** **P < 0.001** ***η***_***p***_^***2***^ **= 0.55**
	***Benguela***	78±9	124±19*^‡^	106±11*^‡^	102±11*^‡^	99±13*^‡^	100±11*^‡^		
	***São Bento***	78±12	145±16*^†^	118±12*^†^	111±11*^†^	109±11*^†^	108±10*^†^		
**RRi (ms)**	***Angola***	783±63	565±77*	659±88*	672±91*	659±86*	655±97*	**F = 109.270** **P < 0.001** ***η***_***p***_^***2***^ **= 0.91**	**F = 4.774** **P < 0.001** ***η***_***p***_^***2***^ **= 0.32**
	***Benguela***	799±77	484±74*^‡^	575±65*^‡^	593±63*^‡^	616±76*^‡^	614±75*		
	***São Bento***	774±77	432±71*^†^	516±62*^†^	546±55*^†^	555±61*^†^	562±56*^†^		
**rMSSD (ms)**	***Angola***	27±13	14±9	18±8	18±9	16±7	16±6	**F = 133.133** **P < 0.001** ***η***_***p***_^***2***^ **= 0.93**	**F = 3.189** **P < 0.01** ***η***_***p***_^***2***^ **= 0.24**
	***Benguela***	27±9	8±5*^‡^	8±5*^‡^	8±5*^‡^	9±6*^‡^	10±5*^‡^		
	***São Bento***	27±8	3±1*^†^	4±2*^†^	4±2*^†^	4±2*^†^	4±3*^†^		
**LF:HF**	***Angola***	6.3±4.0	3.3±1.6	6.0±6.8	6.0±6.7	4.7±3.1	10.3±8.4	**F = 4.489** **P < 0.01** ***η***_***p***_^***2***^ **= 0.31**	**F = 1.480** **P = 0.158** ***η***_***p***_^***2***^ **= 0.13**
	***Benguela***	5.2±2.3	9.1±6.9^‡^	8.8±7.7^‡^	9.9±8.0^‡^	12.3±9.5^‡^	13.2±12.5		
	***São Bento***	4.7±1.8	17.2±17.1	14.6±7.4*^‡^	12.5±5.7*	13.3±10.2^‡^	16.2±10.4		
**SBP (mmHg)**	***Angola***	121±13	154±17*	136±15	128±12	126±10	127±11	**F = 47.989** **P < 0.001** ***η***_***p***_^***2***^ **= 0.82**	**F = 1.138** **P = 0.342** ***η***_***p***_^***2***^ **= 0.10**
	***Benguela***	124±11	161±20*	145±21*	134±13	133±13	133±18		
	***São Bento***	124±14	168±27*	146±17	139±13	130±13	126±16		
**DBP (mmHg)**	***Angola***	81±8	90±11	86±11	84±9	81±8	84±10	**F = 21.460** **P < 0.001** ***η***_***p***_^***2***^ **= 0.68**	**F = 2.572** **P = 0.073** ***η***_***p***_^***2***^ **= 0.20**
	***Benguela***	80±8	100±19	86±7	83±11	86±6	84±7		
	***São Bento***	80±11	108±22*	88±16	84±21	79±10	77±12		
**[Lac] (mM)**	***Angola***	1.2±0.3	6.3±2.4*	6.9±2.9*	5.8±2.7*	5.9±2.5*	5.3±2.4*	**F = 108.620** **P < 0.001** ***η***_***p***_^***2***^ **= 0.92**	**F = 42.157** **P < 0.001** ***η***_***p***_^***2***^ **= 0.82**
	***Benguela***	1.2±0.3	8.3±2.4*^‡^	9.5±3.0*^‡^	9.2±3.0*^‡^	8.7±2.9*^‡^	8.1±2.7*^‡^		
	***São Bento***	1.1±0.2	11.2±2.3*^†^	14.3±2.0*^†^	14.9±2.4*^†^	15.7±2.6*^†^	15.2±2.2*^†^		
**[Gluc] (mM)**	***Angola***	6.5±1.9	5.8±1.2	6.3±1.1	5.5±1.5	6.2±0.8	6.0±1.2	**F = 1.778** **P = 0.135** ***η***_***p***_^***2***^ **= 0.15**	**F = 2.152** **P < 0.05** ***η***_***p***_^***2***^ **= 0.18**
	***Benguela***	5.9±1.3	6.4±0.7	6.5±0.9	6.4±0.8	6.3±0.6	6.3±0.6		
	***São Bento***	6.1±1.1	7.1±0.9^†^	7.7±0.6^†^	7.3±1.4	7.7±1.0^†^	6.8±2.4^†^		
**RPE (pts)**	***Angola***	6.1±0.3	10.0±2.2*	7.7±1.7	8.2±3.9	7.3±2.2	7.5±2.0	**F = 16.729** **P < 0.001** ***η***_***p***_^***2***^ **= 0.62**	**F = 5.855** **P < 0.01** ***η***_***p***_^***2***^ **= 0.36**
	***Benguela***	6.4±0.9	11.0±2.8*	8.8±2.2*	8.1±1.7*	6.9±1.6	7.1±1.8		
	***São Bento***	7.5±1.2	15.5±4.1*^†^	12.5±2.7*^†^	10.5±2.7*^#^	10.0±3.6*^†^	8.6±3.2		
**FS (pts)**	***Angola***	3.6±2.0	2.7±1.7	3.1±1.6	3.4±1.6	3.8±1.5	3.5±1.8	**F = 5.642** **P < 0.01** ***η***_***p***_^***2***^ **= 0.36**	**F = 2.448** **P < 0.05** ***η***_***p***_^***2***^ **= 0.19**
	***Benguela***	3.8±1.7	2.1±1.6	2.8±1.4	3.1±1.6	3.4±1.7	3.1±1.9		
	***São Bento***	3.4±1.9	-0.8±3.0*^†^	0.8±2.4^†^	1.6±2.3^#^	2.2±2.3^‡^	2.6±1.7		
**PA (pts)**	***Angola***	1.0±0.0	3.0±1.2*	2.3±1.6	1.5±0.9	1.6±1.2	1.8±1.5	**F = 17.510** **P < 0.001** ***η***_***p***_^***2***^ **= 0.63**	**F = 3.893** **P < 0.05** ***η***_***p***_^***2***^ **= 0.28**
	***Benguela***	1.2±0.6	3.5±1.6*	2.6±1.4*	2.2±1.5^‡^	2.0±1.5	1.9±1.6		
	***São Bento***	1.0±0.0	4.9±1.5*^‡^	3.9±1.3*^†^	2.8±1.0*^‡^	2.6±1.5^‡^	2.1±1.2		

**HR**: heart rate; **RRi**: absolute mean of R-R interval series; **rMSSD**: root mean square differences of successive RRi; **LF:HF**: sympathetic-vagal balance from the low and high frequency components; **SBP**: systolic blood pressure; **DBP**: diastolic blood pressure; **[Lac]**: blood lactate concentration; **[Gluc]**: blood glucose concentration; **RPE**: rate of perceived exertion; **FS**: feeling scale; **PA**: perceived activation.

*P < 0.05 to Rest

†P < 0.05 to *Benguela* and *Angola*

‡P < 0.05 to *Angola*

#P < 0.05 to *Benguela*.

## Results

Table 2 presents the absolute results of HR_MIN_, HR_AVE_ and HR_PEAK_ during each capoeira performance (*Angola*, *Benguela* and *São Bento*). In addition, these values were relativized for the maximum HR obtained in MAIT (%HR_PPO_). It could be verified a main effect between HR MIN, AVE and PEAK [F(1.19;11.96) = 382.124; P<0.001; η_p_^2^ = 0.97] and interaction HR*performance [F(2.32;23.28) = 26.363; P<0.001; η_p_^2^ = 0.72]. Similar results occurred for the relative values with main effect between HR (%HR_PPO_) MIN, AVE and PEAK [F(1.15;11.52) = 450.407; P<0.001; η_p_^2^ = 0.97] and interaction HR*performance [F(2.30;23.08) = 26.399; P<0.001; η_p_^2^ = 0.72]. Likewise, it could be verified a main effect between RRi in HR MIN, AVE and PEAK [F(1.19;11.88) = 387.527; *P<0*.*001*; η_p_^2^ = 0.97], as well as the interaction RRi*performance [F(2.01;20.17) = 4.004; *P<0*.*01*; η_p_^2^ = 0.28].

Still, it is highlighted that during the execution of the capoeira performances the response of the parasympathetic indicator rMSSD, was statistically different [F(1.152;11.517) = 42.865, P<0.001; η_p_^2^ = 0.81] between the *Angola* (12±4 ms), *Benguela* (8±1 ms) and *São Bento* (3±1 ms) performances. The main time effect also occurred when comparing the responses during the rhythms execution with their respective resting moments [F(1,10) = 143.695; P<0.0001, η_p_^2^ = 0.935], being in *Angola* a resting period of 27±13 ms, in the *Benguela* a resting period of 27±9 ms and in *São Bento* a resting period of 27±8 ms.

Table 3 exhibits the results of physiological and perceptual variables obtained at rest (pre-intervention) and recovery (post-intervention) from different capoeira performances. The two-way ANOVA results with *Post hoc of Bonferroni* are presented in columns on the right side of Table 3.

**Fig 3:**
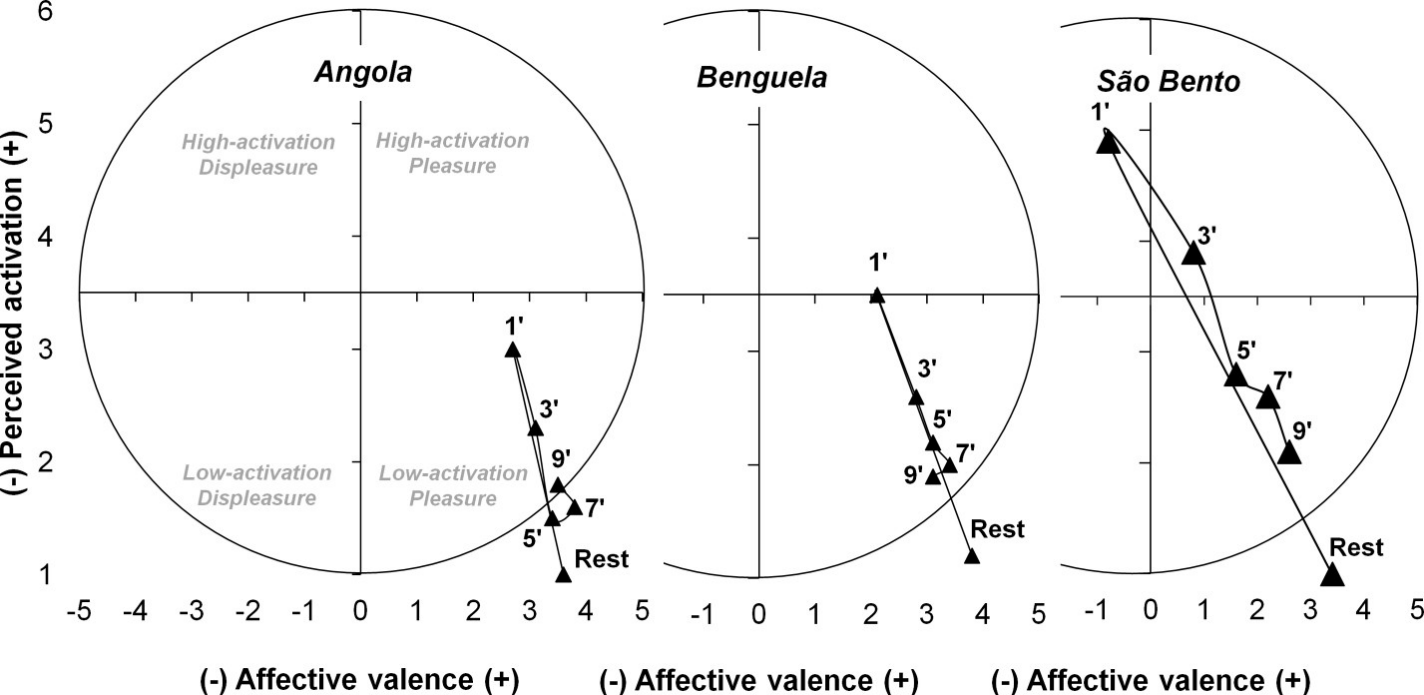
Responses to affective valence (feeling scale) and perceived activation pre and post different capoeira performance plotted in circumplex space [24].

Fig 3 shows the integrated circumflex model of the responses to FS and PA in pre and post-intervention moments for the different capoeira performances. It is noticed that the *Angola* and *Benguela* are found throughout the recovery period on the quadrant corresponding to the basic positive affective bound to a moderate activation state. On the other hand, *São bento* immediately after the intervention of 90 seconds of performance is on the quadrant of negative basic affective and high activation state.

**Fig 4:**
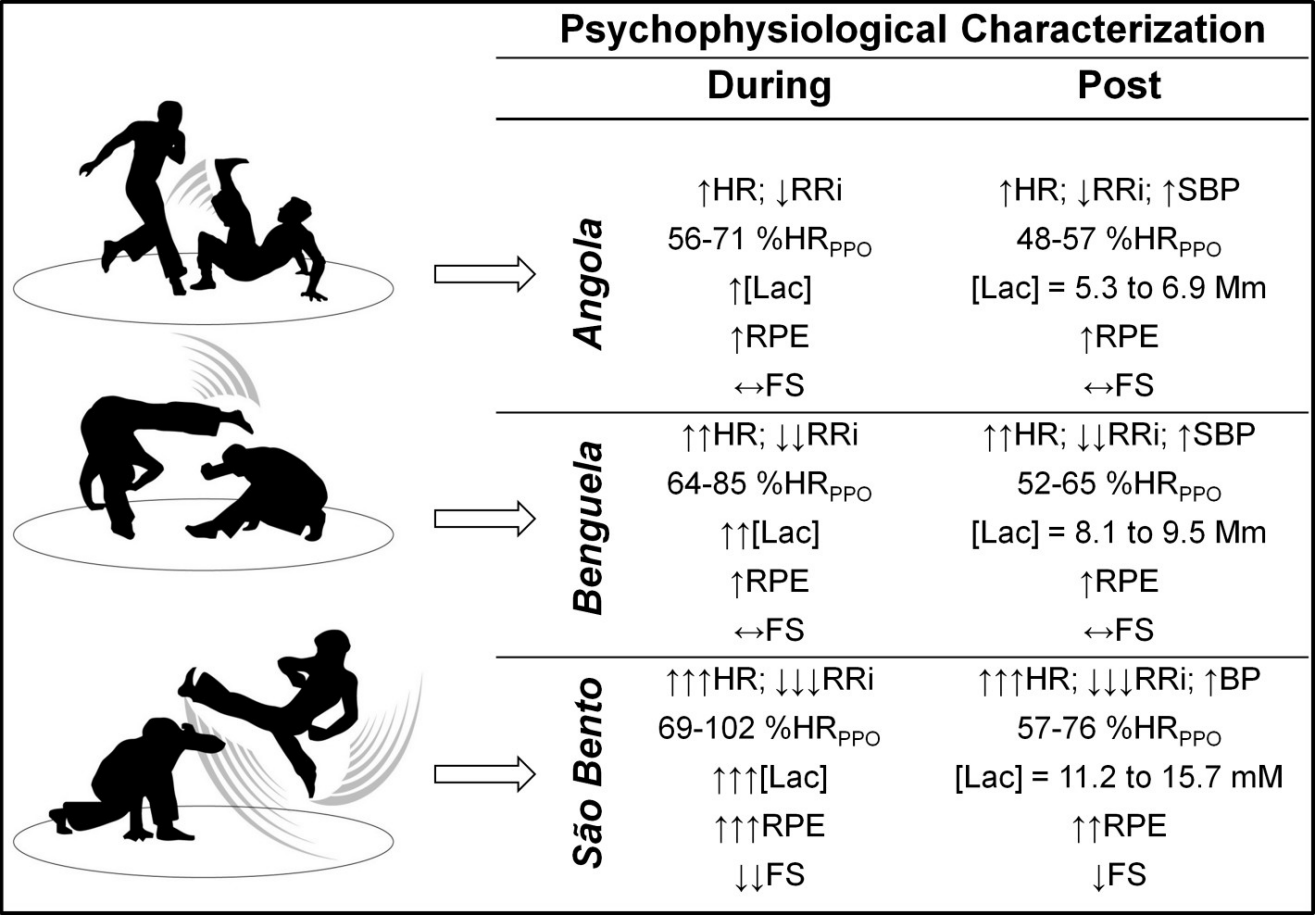
Representative movements of *Angola*, *Benguela* and *São Bento* rhythms and summary of psychophysiological characterization during and right after (1 to 9 minutes) the development of each capoeira performance. The arrows in front of each variable means the magnitude of change due to the capoeira performance.

With the objective to summarize the findings of this study, Fig 4 shows the representative movements with their corresponding speeds in performances of *Angola*, *Benguela* and *São Bento* and their respective psychophysiological responses obtained during and after the intervention in each one of the performances. The arrows before each variable display the magnitude of the adjustment occurred in function of each performance. In addition, for a better understanding of the physiological characterization, areas of numerical results of relative demand (%HR_PPO_) and absolute metabolic ([Lac]) of each rhythm also are presented in Fig 4.

## Discussion

Some studies, within their methodologic particularities, have sought to investigate the physiological responses in karate [25], judo [26], jiujitsu [27], Greco-Roman wrestling [28] and mixed martial arts [29]. The objective of this study was to characterize the psychophysiological demand of different capoeira performances on male individuals experienced in the modality. The main finding was that the *São Bento* rhythm resulted in a greater physiological demand according to the HR, [Lac] and cardiac autonomic indicators, with the *Benguela* rhythm being intermediate while the *Angola* rhythm presented a reduced demand. The [Gluc] increased after the *São Bento* rhythm when compared to the other rhythms. Furthermore, the perceptual demand presented increased in terms of RPE and PA variables and decreased in terms of FS when the *São Bento* performance was analyzed in comparison to the *Angola* and/or *Benguela* in capoeira.

The general characteristics presented in Table 1 reveal that studied sample meets the health criteria for body mass index, waist circumference, body fat, BP [8] and [Gluc] [30]. Furthermore, the aerobic fitness of the participants, as evaluated by PPO_MAIT_ (Table 1), is in agreement to previous studies with physically active and health adults [31].

Regarding the BP adjustments, only one study verified such responses after different capoeira performances [5]. The results of this study corroborate the findings of the study herein, in which no significant differences were observed between the performances immediately after their execution in terms of SBP and DBP (Table 3). On the other hand, unlike the results of Silva et al. [5], significant differences in SBP immediately after each performance and DBP immediately after the *São Bento* rhythm occurred in comparison to their respective resting period (Table 3).

Analysis of the cardiovascular system indicated significant differences in HR among the capoeira performances during both the implementation (Table 2) and recovery times (Table 3). In the same way, important differences were evidenced in HR when comparing the recovery from each rhythm with the respective resting moments (Table 3). Silva et al. [5,10], who investigated the HR in similar performances, did not find the same responses as in the present study, possibly due to methodological differences in the experimental design proposed. Previous studies have shown that HR had a nonlinear relationship with oxygen consumption during activities with intermittent characteristics [32]. Therefore, the different HR responses evidenced in capoeira (Table 2) can be explained mainly by the specific movement requirements for each rhythm associated with the intermittent muscular contraction of the movement. Regardless of the relationship between HR and oxygen consumption in intermittent activities such as in capoeira, it is speculated that with the *São Bento* rhythm, the oxygen consumption is higher, followed by the *Benguela* rhythm and, with lower demand, the *Angola* rhythm. Furthermore, it is speculated that the mechanistic pathway that explains the HR response as a function of the capoeira performance execution is adjusted during the performance from a vagal withdrawal [33,34], with consequent increase of the sympathetic nervous activity [33], which can be observed from the RRi (Table 2) and rMSSD (parasympathetic indicator) reduction that occurred in the study herein. Such autonomic response leads to an increase in HR in proportion to the demand of exercise involved in *Angola*, *Benguela* and *São Bento* capoeira performances (Table 2).

From a clinical point of view, it is worth pointing out possible implications in specifically adopting the *São Bento* performance because this has shown to be characterized by a maximum intensity domain (Table 2). Immediately after the execution of *São Bento* performance, the SBP and DBP values were higher compared to during their resting moments. In the same way, even during the seven minutes of recovery from the *São Bento* execution, the [Gluc] was higher when compared to the *Benguela* and *Angola* performances (Table 3). These results suggest caution in applying intense capoeira performance for populations with cardiovascular risk factors such as hypertension and diabetes because the demand for increased intensity (Table 2) may suggest increased recovery times in the parasympathetic modulation (rMSSD) with consequent increased adrenergic discharge and increases in BP and [Gluc] in the practitioner (Table 3). Other studies have shown that the [Gluc] increases again from the lactate threshold measured in aerobic exercise [16] and conventional resistance exercise [35] in samples of individuals with type-2 diabetes. Following the same reasoning, a single session of resistance exercise when performed at intensities below a lactate threshold previously identified resulted in better glycemic control in individuals with type-2 diabetes when compared to a session performed above the lactate threshold also [36]. These authors, based on theoretical assumptions in the literature [11], have suggested that the lactate threshold in both aerobic and resistance exercise delineates a point of balance between the uptake and endogenous production of glucose.

Others authors [37] have demonstrated that RPE 13 and 14 on the Borg scale of 15 points [6 to 20] delineate an intensity of anaerobic threshold obtained by ventilatory variables, [Lac] and [Gluc]. In the study herein, the *Angola* performance reflected an RPE equal to 10.0 ± 2.2, while the *Benguela* reflected an RPE equal to 11.0 ± 2.8 and *São Bento* an RPE equal to 15.5 ± 4.1. These findings, along with the responses of [Lac] (Table 3), strengthen the different domains of intensities in the performances of this modality, especially the superior intensity of *São Bento* capoeira performance.

Furthermore, the study herein evaluated the basic affective (FS) effect of different rhythms, and unlike the *Angola* and *Benguela* performances, which feature lower intensities and adjust positively to FS, *São Bento* resulted in negative FS immediately after its completion (Table 3). Ekkekakis et al. [38] demonstrated that exercise intensities above the ventilatory threshold or even progressive increases in exercise intensity cause a decline in basic affective effects with a consequent negative response in the FS. However, such responses are reversible in exercise recovery moments [39], as can be seen in terms of the *São Bento* rhythm from the third minute of recovery (Table 3). These findings may have important practical applications for capoeira instructors who have either goals of controlling training loads from instruments of easy application, such as the perceptual scales (FS and RPE), or goals in terms of individuals’ adherence to training because acute responses in the FS show an important association with adherence to an exercise program [40].

Even with a significant RPE increase right after the execution of *Angola* and *Benguela* performances compared to the resting moment, the basic affective (FS) effect remained positive throughout the recovery period in these rhythms (Table 3). Fig 3 exhibits the perceptual behavior, integrating the dimension of the affective space [24]. It was observed that the investigated recovery within the rhythms reflects a positive basic affective effect (low activation-pleasure), except for the *São Bento* performance, which was on a negative quadrant (high activation-displeasure) only in the first minute of recovery. It is worth highlighting that the movements carried out by the practitioner in the different capoeira rhythms are self-selected. The self-selection of movements tends to be associated with a greater functional justification for the task executor, which redirects the internal attention focus of muscle contraction—a more evident aspect in the imposed conventional exercise—to the external attention focus from established purposes of entertainment, play and challenge [14]. This condition can operate in the perceptual responses during the execution of a 60-minute capoeira class, where in its final part, self-selected movements (in pairs of participants) are promoted, and even with the RPE and HR increasing when compared with imposed movements (performing individual *ginga*), the FS was maintained without differences and in positive affective domains [41]. These results, together with the findings of the present study, suggest capoeira, especially with self-selected movements, as a possible hedonic model of physical activity prescription, which aims at adherence of an individual to a regular physical exercise program [13,14] that does not promote feelings of pain and distress in the practitioner [15]. However, a novel longitudinal study for this purpose needs to be developed.

The knowledge of the psychophysiological demand of *Angola*, *Benguela* and *São Bento* can provide information to the practitioner and coach to conduct specific physical training strategies with goals of performance improvement. For example, the [Lac] as a result of physical sports performance has been considered an important anaerobic metabolic indicator of the energy systems in judo [42] and anaerobic glycolytic capacity, with its production being directly related to better performance in races of maximum 100, 200 and 400 meters performed by athletes of various ages [43]. In this sense, the observation of HR and [Lac] responses in capoeira suggests training stimuli targeting specific overload in the glycolytic pathway to enhance performance in *São Bento*, mixed glycolytic-oxidative stimuli for performance in *Benguela* and, finally, oxidative stimuli to improve performance in *Angola*.

On the other hand, it is important to highlight that while analyzing the HR_AVE_ and HR_PEAK_ absolute and relative values during the execution of the different capoeira performances (Table 2), demands of exercise intensity recommended by the *American College of Sports Medicine* for the promotion of positive cardiovascular adaptations to the health and performance of an individual were verified [9]. The different capoeira performances may be classified in domains from moderate intensity, starting with *Angola* performance, to vigorous intensity in *Benguela* performance and maximum intensity in *São Bento* performance. Alternatively, based on this knowledge and with the function of the proposed objectives by the practitioner, the capoeira instructor can guide the training program, both in a specific way using only one type of rhythm of play, as well as by combining the different rhythms and even intercalating them within the same capoeira training session.

The results of the present study can be applied in sports medicine and in performance directed at capoeira. The practical significance of results in a clinical perspective demonstrates that different capoeira rhythms modulate cardiometabolic variables distinctly, bringing important implications in the capoeira prescription for populations with cardiovascular diseases risk factors. A high-intensity demand, as demonstrated in the *São Bento* rhythm, may reflect important autonomic modulation with a consequent increase in BP and [Gluc] responses. On the other hand, capoeira training responses are relevant because previous studies demonstrated cardiovascular [6] and neuromuscular [7] benefits after ten (1x/week) and eight (2x/week) weeks, respectively. Additionally, in a practical perspective, the capoeira psychophysiological characterization enables guiding training aimed at improving performance in the rhythms. The HR and [Lac] responses suggest training stimuli targeting specific overload in the glycolytic pathway to enhance *São Bento* performance, mixed glycolytic-oxidative stimuli for the *Benguela* performance and oxidative stimuli to improve the *Angola* performance. In addition, the results showed that the *Angola* (moderate intensity), *Benguela* (vigorous intensity) and *São Bento* (maximum intensity) rhythms require important cardiovascular demand, which can promote positive adaptations in both aspects related to sports performance and the health of the practitioner [9].

One of the main limitations of this study was the lack of oxygen consumption measurement during the execution of the different capoeira performances. Such a procedure was not possible due to the motor movements required for the performer in each performance; movements with extreme degrees of flexibility in the regions of the lower limbs, upper limbs and trunk; explosions in the coups; jumps; changes of direction and positions reversed are needed in capoeira practice and make the use of more complex equipment coupled with the body of the practitioner unviable. However, this is the first study to integrate psychophysiological responses in the functions of different capoeira performances, which were performed on different days, in a randomized order and with pairs of experienced individuals matched with the execution of the each rhythm, strengthening the internal validity of the results obtained. Another limitation was the variation of instrument players within the same capoeira rhythm between the different pairs of participants (e.g., in *São Bento*). However, the musical instruments were played by experienced individuals, keeping the same players for each performance (pairs of participants), and the songs were also sung in a standardized way for each performance.

## Conclusion

We concluded that different capoeira performances modulate distinctively psychophysiological variables during performance and recovery. The *São Bento* rhythm resulted in a greater physiological demand according to the HR, [Lac] and cardiac autonomic indicators (RRi and rMSSD reduction), with the *Benguela* rhythm being intermediate while the *Angola* rhythm presented a reduced demand. Still, the [Gluc] increased after the *São Bento* rhythm when compared to the other rhythms (*Angola* and *Benguela*). Finally, the perceptual demand presented increased in terms of RPE and PA variables and decreased in terms of FS when the *São Bento* performance was analyzed in comparison to the *Angola* and/or *Benguela* in capoeira.

## Supporting information

S1 File CONSORT_Checklist. (DOC)
